# Safety of intravenous administration of hydrogen-enriched fluid in patients with acute cerebral ischemia: initial clinical studies

**DOI:** 10.1186/2045-9912-3-13

**Published:** 2013-06-25

**Authors:** Kimihiro Nagatani, Hiroshi Nawashiro, Satoru Takeuchi, Satoshi Tomura, Naoki Otani, Hideo Osada, Kojiro Wada, Hiroshi Katoh, Nobusuke Tsuzuki, Kentaro Mori

**Affiliations:** 1 Department of Neurosurgery, National Defense Medical College 3-2 Namiki, Tokorozawa, Saitama, 359-8513, Japan; 2 Department of Neurosurgery, Ken-o-Tokorozawa Hospital, Higashisayamagaoka, Tokorozawa, Saitama, 4-2692-1, Japan; 3 Department of Neurosurgery, Kuki General Hospital, 418-1 Kamihayami, Kuki, Saitama, Japan

**Keywords:** Acute ischemic stroke, Edaravone, Free radical scavenger, Hydrogen, Reactive oxygen species, Safety, Tissue plasminogen activator

## Abstract

**Background:**

Most of the results regarding hydrogen (H_2_) therapy for acute cerebral ischemia are derived from *in vitro* studies and animal experiments, with only a few obtained from human trials with a limited number of subjects. Thus, there is a paucity of information regarding both the beneficial therapeutic effects as well as the side effects of H_2_ on acute cerebral ischemia in humans. We designed a pilot study to investigate single dose intravenous H_2_-administration in combination with edaravone, aiming to provide an initial estimate of the possible risks and benefits in select patients presenting with acute ischemic stroke.

**Methods:**

An open-label, prospective, non-randomized study of intravenous H_2_-administration was performed in 38 patients hospitalized for acute ischemic stroke. All patients received an H_2_-enriched intravenous solution in addition to edaravone immediately after the diagnosis of acute ischemic stroke. Acute stroke patients within 3 h of onset received intravenous tissue plasminogen activator (t-PA) (0.6 mg/kg) treatment, and patients receiving t-PA had to commence the administration of the H_2_-enriched intravenous solution and edaravone before or at the same time as the t-PA was infused.

**Results:**

Complications were observed in 2 patients (5.3%), which consisted of diarrhea in 1 patient (2.6%) and cardiac failure in 1 patient (2.6%). No deterioration in laboratory tests, urinary tests, ECG, or chest X-ray radiograms occurred in any patient in this study. In all patients, the mean National Institutes of Health Stroke Scale (NIHSS) scores at baseline, and 7, 30, and 90 d after admission were 8.2 ± 7.5, 5.6 ± 7.1, 4.9 ± 6.5, and 4.5 ± 6.3, respectively. The early recanalization was identified in 4 of 11 patients (36.4%) who received intravenous t-PA administration. Hemorrhagic transformation was observed in 2 patients (18.2%). None of the patients in this study that were treated with t-PA developed symptomatic intracranial hemorrhage.

**Conclusions:**

Data from the current study indicate that an H_2_-enriched intravenous solution is safe for patients with acute cerebral infarction, including patients treated with t-PA.

## Background

Reactive oxygen species (ROS) have been implicated in brain injury after ischemic stroke [1]. The dominant ROS (hydroxyl radicals and peroxynitrite) indiscriminately react with nucleic acids, lipids, and proteins, resulting in DNA fragmentation, lipid peroxidation, and protein inactivation [2]. Among the ROS, hydroxyl radicals and peroxynitrite appear to play a critical role in tissue injury via reactions with nucleic acids, lipids, and proteins [3]. The human body has no endogenous detoxification system for hydroxyl radicals and peroxynitrite [3].

Recently, Ohsawa et al. revealed that hydrogen (H_2_) gas selectively scavenges hydroxyl radicals and peroxynitrite in vitro, thereby exerting a therapeutic antioxidant activity in a focal cerebral ischemia and reperfusion (I/R) rat model [3]. They reported that H_2_ was more effective than edaravone, which was approved in Japan as an ROS scavenger for the treatment of cerebral infarction. Moreover, several studies claim that H_2_ mediates cellular protection thorough attenuation of production of these dominant ROS following acute cerebral ischemia [4-6]. Most of the results regarding H_2_ therapy in acute cerebral ischemia come from *in vitro* studies and animal experiments, with only a few derived from human trials that involved a limited number of subjects [5,7]. Thus, more information is needed concerning the beneficial effects and side effects of H_2_ as a treatment for acute cerebral ischemia in humans. For these reasons, we designed a pilot study in which single dose intravenous H_2_-administration in combination with edaravone was given within 72 h after onset, aiming to provide an initial estimate of the possible risks and benefits in select patients presenting with acute ischemic stroke.

## Methods

### Patients

An open-label, prospective, non-randomized study of intravenous H_2_-administration was performed at 3 institutions in Japan (the National Defense Medical College, the Kuki General Hospital, and the Ken-o-Tokorozawa Hospital) from July 2011 through December 2012. The Clinical Research Ethics Committee of these institutions approved this study. Prior informed consent was obtained from all study participants or their relatives.

Patients were eligible for enrollment if they were 18 years or older and had a clinical diagnosis of acute ischemic stroke within 72 h of symptom onset. All patients underwent examination using computed tomography (CT) or magnetic resonance imaging (MRI) immediately after admission. A diagnosis of stroke was based on clinical findings. Baseline data (age, sex), conventional vascular risk factors (hypertension, diabetes mellitus, hyperlipidemia), and past history of smoking, atrial fibrillation, ischemic stroke and hemorrhagic stroke were recorded. Patients whose pertinent data could not be evaluated at the time of stroke onset were excluded from this study. Based on the location of the ischemic lesions, the patients were divided into 2 groups [8]. In Group I (GI) patients, the infarct was located in cortical regions within the cerebral hemisphere and involved the frontal, parietal, and temporal lobe or the occipital lobe and cerebellum. In Group II (GII) patients, the infarct involved basal ganglia regions in the anterior circulation (putamen, caudate head), corona radiate, or brain stem and thalamus. The stroke subtypes were defined according to the Trial of Org 10172 in Acute Stroke Treatment (TOAST) classification system [9]. Of the 38 patients, 12 (31.6%) had cardioembolic infarcts, 14 (36.8%) had atherothrombotic infarcts, 11 (28.9%) had lacunar infarcts, and 1 (2.6%) had a stroke of undetermined etiology. The atherothrombotic infarct group included patients with clinical and imaging findings of either significant stenosis or occlusion of a major artery or branch of the cortical artery, due to atherosclerosis. The cardioembolic infarct group included patients with arterial occlusion due to an embolus arising from the heart. The lacunar infarction group included patients with one of the traditional clinical lacunar syndromes and no evidence of cerebral cortical dysfunction, and patients whose MRI did not show any lesions exceeding 1.5 cm in diameter.

### Treatment

There was no control group in this study. All patients received intravenous H_2_-enriched glucose-electrolyte solution in addition to edaravone immediately after the diagnosis of acute ischemic stroke. For all patients, intravenous edaravone (a 30 mg Edaravone Kit) was given twice a day (every 12 h), and H_2_-enriched intravenous solutions (200 ml) were added at the speed of 200 ml/h twice a day (every 12 h). All patients received appropriate routine stroke care. Acute stroke patients within 3 h of onset received intravenous t-PA treatment (0.6 mg/kg), and those patients receiving t-PA had to commence the administration of H_2_-enriched intravenous solution and edaravone before or at the same time that the t-PA was infused.

### Production of an H_2_-enriched glucose-electrolyte solution

An H_2_-enriched glucose-electrolyte solution was produced using a non-destructive hydrogen adding apparatus (Miz Co., Fujisawa, Japan; Patent No.4486157, Patent Gazette of Japan 2010) as has been reported elsewhere [10]. Bags of glucose-electrolyte solution (Soldem 1, 200 ml/bag, Terumo, Tokyo, Japan) were immersed, without opening or altering the bag, in a water tank in which water is electrolyzed periodically to produce water with hydrogen concentrations of up to 1.6 ppm. The concentration of hydrogen in the bag reached saturation, increasing to more than 1.0 ppm because of diffusion through the wall of the bag. Additional information describing this process can be found at: http://www.e-miz.co.jp/english/technology.html#non_destructive.

### Clinical diagnosis and evaluation

An NIHSS score was assigned upon admission to the hospital, and 7, 30, and 90 d after admission to evaluate neurological deficits. Patients were also evaluated using a modified Rankin scale (mRS) (7, 30, and 90 d after admission) and the Barthel Index (7, 30, and 90 d after admission). Vital signs were recorded at enrollment and at specified times throughout the infusion and follow-up periods. Venous blood sampling for the malondialdehyde (MDA)-modified low-density lipoprotein (LDL) was taken as a serum-based indicator of oxidative stress [11]. Other biochemical analyses, urine sampling, 12-lead electrocardiography (ECG), and plain chest X-rays were performed at the time of enrollment, and on the 7th day after admission, and were analyzed centrally.

Prior to administration of t-PA, 3D-CT angiography (3DCTA) or MR angiography (MRA) were performed to identify the occluded arteries. Follow-up 3DCTA or MRA was performed 24 h after t-PA treatment to identify the presence or absence of early recanalization in the occluded arteries. Recanalization was graded as complete, partial, or no recanalization according to a previous report [12], as follows: 1) complete recanalization: reappearance of the entire occluded artery and the distal branches of vessels; 2) partial recanalization: restoration of part of the distal vessel supplied by the occluded artery; and 3) no recanalization: persistent occlusion. To assess any effect of H_2_ on hemorrhagic transformation after t-PA administration, brain imaging was repeated after 24 h in patients who were receiving concomitant treatment with t-PA. Symptomatic hemorrhagic transformation was defined as an increase of or more 4 points according to the NIHSS score that occurred within 24 h, and evidence of any blood on neuroimaging performed 24 h after treatment with t-PA.

### Statistical analysis

All data are presented as the mean ± SD. All analyses were performed using Student’s t test for paired data. A value of P < 0.05 was considered statistically significant. The GraphPad Prism 4.0 software program (San Diego, CA, USA) was used for all statistical tests.

## Results

A total of 38 patients were enrolled in the study: 25 at the National Defense Medical College, 10 at the Kuki General Hospital, and 3 at the Ken-o-Tokorozawa Hospital. The demographics and baseline characteristics of the patients are provided in Table 1. They were 27 men and 11 women, ranging in age from 40 to 95 yr (69.4 ± 10.7). Of the 38 patients, 27 were GI patients and 11 were GII patients. The mean baseline NIHSS score in total, GI, and GII patients was 8.2 ± 7.5, 10 ± 8.2, and 3.9 ± 2.4, respectively. The mean time (h) from onset of symptoms to initiation of treatment in total, GI, and GII patients was 16 ± 18.1, 14.9 ± 18.7, and 18.8 ± 16.3, respectively. The mean duration (d) of treatment with edaravone and hydrogen in total, GI, and GII patients was 10.8 ± 3.4, 10.7 ± 3.5, and 11.1 ± 3, respectively.

**Table 1:** Demographics and baseline characteristics of patients recruited for this study

		**Total**	**Group I**	**Group II**
		**n = 38**	**n = 27**	**n = 11**
Male gender, n (%)		27 (71)	19 (70)	8 (73)
Age, years		69.4 ± 10.7	70.9 ± 11.2	65.5 ± 7.9
Past history, n (%)				
	Hypertension	25 (66)	19 (70)	6 (55)
	Diabetes mellitus	16 (42)	9 (33)	7 (64)
	Hyperlipidemia	7 (18)	4 (15)	3 (27)
	Smoking	8 (21)	4 (15)	3 (27)
	Atrial fibrillation	11 (29)	11 (41)	0 (0)
	Ischemic stroke	11 (29)	9 (33)	2 (18)
	Intracerebral hemorrhage	2 (5)	1 (4)	1 (9)
	Subarachnoid hemorrhage	0 (0)	0 (0)	0 (0)
Infarct side, n (%)				
	Right	20 (53)	13 (48)	7 (64)
	Left	18 (47)	14 (52)	4 (36)
Baseline NIHSS score, mean		8.2 ± 7.5	10 ± 8.2	3.9 ± 2.4
Interval from onset to treatment with edaravone and hydrogen, h		16 ± 18.1	14.9 ± 18.7	18.8 ± 6.3
Duration of treatment with edaravone and hydrogen, d		10.8 ± 3.4	10.7 ± 3.5	11.1 ± 3
Laboratory data				
	HbA1C,%	6.3 ± 1.7	6 ± 1.4	7 ± 2
	BG, mg/dl	145.2 ± 58.2	134 ± 48.9	171.6 ± 68.8
	TG, mg/dl	116.7 ± 61.3	121 ± 67.7	107.8 ± 43.7
	TC, mg/dl	194.3 ± 43.4	186.6 ± 38.3	217.3 ± 49.4
	LDL, mg/dl	114.3 ± 33.7	109 ± 25.7	126.5 ± 44.7
	UA, mg/dl	5.3 ± 1.4	5.2 ± 1.4	5.6 ± 0.9
	PT-INR	1.14 ± 0.3	1.2 ± 0.3	1.1 ± 0.06
	APTT, seconds	30.7 ± 3.9	31 ± 4.1	30.1 ± 3.2
	Fibrinogen, mg/dl	336.7 ± 101.9	333.6 ± 107.4	349 ± 74.9
	Hct (%)	41.3 ± 6.4	40.7 ± 6.7	42.8 ± 5.3

*BG*, blood glucose; *TG*, triglyceride; *TC*, total cholesterol; *LDL*, low-density lipoprotein; *UA*, uric acid; *PT-INR*, prothrombin time-international normalized ratio; *APTT*, activated partial thromboplastin time; *Hct*, hematocrit.

### Safety/adverse events

Complications were observed in 2 patients (5.3% of all patients) (Table 2). Such complications consisted of diarrhea in 1 patient (2.6%) and cardiac failure in 1 patient (2.6%). In the patient who had cardiac failure, the administration of H_2_-enriched intravenous solution and edaravone was stopped on the 9th day after admission, in order to avoid volume overload. No deterioration in laboratory tests or ECG occurred in any patients in this study. Analysis of urine sampling and plain chest X-rays showed no indications of pulmonary or urinary tract infection induced by the administration of the H_2_-enriched intravenous solution.

**Table 2:** Complications experienced by patients recruited for this study

		**Total (n = 38)**	**G I (n = 27)**	**G II (n = 11)**
Complication, n (%)		2 (5.3)	2 (7.4)	0 (0)
	Diarrhea	1 (2.6)	1 (3.7)	0 (0)
	Cardiac failure	1 (2.6)	1 (3.7)	0 (0)

### Changes in the level of MDA-LDL, a serum marker for oxidative stress

**Figure 1:**
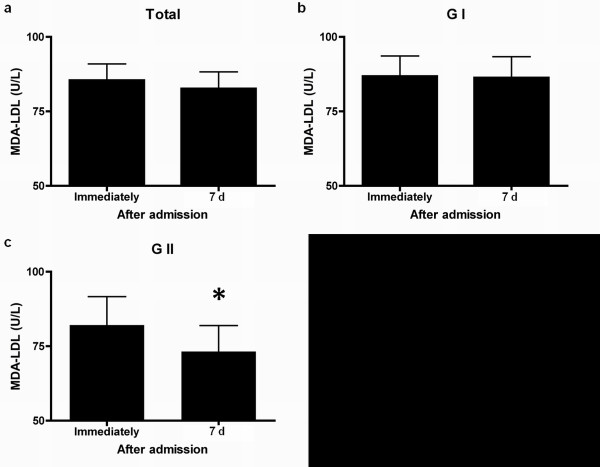
**Changes in the levels of the serum oxidative stress marker MDA-LDL (malondialdehyde-modified LDL).** Compared to pretreatment levels, the level of serum MDA-LDL 7 d after admission decreased, but did not differ significantly from that of pretreatment levels in all patients (**a**) and GI patients (**b**), respectively (85.4 ± 33.5 vs. 82.6 ±31.5, 86.8 ± 34.7 vs. 86.3 ±33.1). By contrast, in comparison with pretreatment levels, GII patients (**c**) showed a significant decrease in the MDA-LDL level 7 d after admission (82.1 ± 30.2 vs. 73.2 ± 24.6, *P = 0.0111). Values are expressed as mean ± SD.

Compared to pretreatment levels, the level of serum MDA-LDL on the 7th day after admission decreased, but did not differ significantly from that of pretreatment in all patients or in GI patients alone, respectively (85.4 ± 33.5 vs. 82.6 ± 31.5, 86.8 ± 34.7 vs. 86.3 ± 33.1) (Figure 1, Table 3). By contrast, in comparison with pretreatment levels, GII patients showed a significant decrease in MDA-LDL levels 7th day after admission (82.1 ± 30.2 vs. 73.2 ± 24.6, P = 0.0111) (Figure 1, Table 3).

**Table 3:** Serum MDA-LDL levels in patients recruited for this study

	**Immediately after admission**	**7 d after admission**	**P**
Total (n = 38), U/L	85.4 ± 33.5	82.6 ± 31.5	0.3424
G I (n = 27), U/L	86.8 ± 34.7	86.3 ± 33.1	0.9262
G II (n = 11), U/L	82.1 ± 30.2	73.2 ± 24.6	0.0111

*MDA-LDL*, malondialdehyde modified-low density lipoprotein.

### Outcomes of patients

The outcomes of patients recruited in this study are shown in Table 4. In all patients, the mean NIHSS scores 7, 30, and 90 d after admission were 5.6 ± 7.1, 4.9 ± 6.5, and 4.5 ± 6.3, respectively. The mean Barthel index 7, 30, and 90 d after admission, in all patients was 63.8 ± 39.8, 67.6 ± 40.3, and 69.3 ± 39.8, respectively. In all patients (n = 38), the rate of assignment of grades 4–6 on the mRS, assessed at 7, 30, and 90 d after admission was 39.5 (15/38), 39.5 (15/38), and 34.2% (13/38), respectively. One of the 38 patients (2.6%) died within 90 d after admission.

**Table 4:** Outcomes of patients recruited for this study

		**Total (n = 38)**			**G I (n = 27)**			**G II (n = 11)**		
		**7 d after admission**	**30 d after admission**	**90 d after admission**	**7 d after admission**	**30 d after admission**	**90 d after admission**	**7 d after admission**	**30 d after admission**	**90 d after admission**
NIHSS score, mean		5.6 ± 7.1	4.9 ± 6.5	4.5 ± 6.3	7.2 ± 7.8	6.4 ± 7.2	6.0 ± 6.9	1.8 ± 1.1	1.4 ± 1.6	1.1 ± 1.4
mRS, n (%)										
	4-6	15 (39.5)	15 (39.5)	13 (34.2)	14 (51.8)	13 (48.1)	12 (44.4)	1 (9.1)	2 (18.2)	1 (9.1)
	Death	1 (2.6)	1 (2.6)	1 (2.6)	1 (3.7)	1 (3.7)	1 (3.7)	0 (0)	0 (0)	0 (0)
Barthel index, mean		63.8 ± 39.8	67.6 ± 40.3	69.3 ± 39.8	55 ± 42.9	58.3 ± 43.2	59.6 ± 42.8	84.5 ± 19.0	89.5 ± 19.4	92.3 ± 15.9
Patients with t-PA infusion										
		n = 11			n = 10			n = 1		
Early recanalization, n (%)		4 (36.4)			4 (40)			0 (0)		
	Complete	1 (9.1)			1 (10)			0 (0)		
	Partial	3 (27.3)			3 (30)			0 (0)		
Hemorrhagic transformation, n (%)		2 (18.2)			2 (20)			0 (0)		
Symptomatic, n (%)		0 (0)			0 (0)			0 (0)		

In total, 11 patients were administered intravenous t-PA (Table 4). Early recanalization was identified in 4 patients (36.4%) (complete in 1 patient (9.1%), and partial in 3 patients (27.3%)). Hemorrhagic transformation was observed in 2 patients (18.2%). In this study, none of the patients treated with t-PA developed symptomatic intracranial hemorrhage.

## Discussion

The present study shows that 2 patients (5.3%) had experienced complications resulting from the treatment with H_2_-enriched fluid, consisting of diarrhea in 1 patient, and cardiac failure in 1 patient, through 90 d after admission. No deterioration in laboratory tests, urinary tests, ECG, or chest X-rays occurred in any patients in this study. To the best of our knowledge, this is the largest case series of patients with ischemic cerebral infarcts treated with an H_2_-enriched intravenous solution. To date, this report also represents the largest case series with the longest duration of follow-up.

As for the adverse effects of hydrogen therapy in humans, Nakao et al. reported that 6 adverse events, experienced by 4 people (20%) receiving H_2_-supplemented drinking water, were assessed by the investigator as having a possible relationship to hydrogen exposure [13]. In their report, these adverse events included loose stools (in 3 of 20 patients), increased frequency of bowel movement (1 patient), heartburn (1 patient) and headache (1 patient). We also observed diarrhea in 1 patient (2.6%) in this study. Moreover, Ono et al. recently reported that administration of intravenous H_2_-enriched saline to 8 patients with acute brainstem infarction caused no adverse effects [7]. According to their report, H_2_-enriched intravenous saline (250 ml) was added for 8 patients twice a day for 7 d, therefore, their dose of H_2_-administration was similar to that of our study. On the basis of these reports, we considered that treatment using an H_2_-enriched intravenous solution is reasonably safe for patients with acute cerebral infarction. In this study, 1 patient (2.6%) who had a history of atrial fibrillation experienced cardiac failure following treatment with H_2_-enriched fluid. However, this adverse effect was hypothesized to be due to volume overload. Further studies that compare patients with and without H_2_ treatment are needed to confirm the safety of intravenous H_2_-administration in patients with acute cerebral infarction.

Our study showed that the level of serum MDA-LDL 7 d after admission did not differ significantly from that of pretreatment levels in GI patients. However, in this study, GII patients showed a significant decrease in the MDA-LDL level 7 d after admission, in comparison with pretreatment levels. Uno et al. reported that patients with atherothrombotic and cortical infarcts manifested significantly higher plasma-oxidized low-density lipoprotein (OxLDL) levels as an oxidative stress marker, compared to patients with lacunar infarcts [14]. Plasma OxLDL levels were correlated with the infarct volume in patients with acute cerebral infarction [15]. On the basis of these reports, we concluded that the amount of oxidative stress produced in GI patients was too much to be scavenged by the treatment with edaravone and H_2_-enriched intravenous solution in this study, although its antioxidative effect was sufficient to significantly reduce oxidative stress in GII patients. We believe that further studies that compare patients with and without H_2_ treatment are needed to elucidate the extent to which H_2_ reduces oxidative stress, and to optimize the dose for H_2_ treatment in patients with acute cerebral infarction. As for the functional outcome of patients in this study, it appeared that the increased efficacy of treatment with H_2_-enriched intravenous solution together with edaravone was not obvious, compared to the previous reports of patients with acute cerebral infarction treated by intravenous edaravone alone [16]. Others have reported that combined edaravone and H_2_-administration reduces oxidative damage, thereby limiting the degree of ischemic brain damage, and improving neurological outcomes [3,6,8,16]. Therefore, we concluded that the antioxidative effect obtained from the treatment protocol in this study might be not sufficient to improve neurological outcomes in our patients. Further investigations are required in order to draw conclusions regarding the clinical efficacy of H_2_-administration in patients with acute cerebral infarction.

Cerebral I/R induced by t-PA treatment results in disruption of the blood–brain barrier (BBB), and causes hemorrhagic transformation because ROS produced during I/R results in damage to vascular endothelial cells and the basement membrane [17,18]. Thus, the inhibition of ROS is an effective therapeutic intervention for improving the integrity of the BBB during I/R injury [6,19]. Our results showed that in patients treated with t-PA infusion, the early recanalization was identified in 4 patients (36.4%). Our data also showed that hemorrhagic transformation was observed in 2 patients (18.2%) and that not a single patient who was treated with t-PA developed symptomatic intracranial hemorrhage. Kimura et al. previously reported that early recanalization within 1 h after t-PA infusion (0.6 mg/kg, the same dose used in our study) was observed in 15 out of 40 acute stroke patients (37.5%) treated with edaravone, and that hemorrhagic transformation 24 h after t-PA infusion occurred in 25 of 40 patients (62.5%) [12]. Symptomatic intracranial hemorrhage was observed in 2 of 40 patients (5%) in their study. While we cannot make a simple comparison of our results with results obtained in their study, we believe that H_2_-administration with edaravone was not harmful to patients treated with t-PA, and that H_2_-administration with edaravone might ameliorate hemorrhagic transformation via reduction of oxidative stress. To our knowledge, the present study is the first to report H_2_-administration with t-PA in patients diagnosed with acute ischemic stroke. However, further studies comparing patients with and without H_2_ treatment in large populations are needed to confirm the safety of intravenous H_2_-administration in patients treated with t-PA. Moreover, additional studies are required to draw conclusions regarding the clinical effectiveness of H_2_-administration in patients treated with t-PA.

We are aware that the number of cases enrolled was too small to draw definite conclusions about the efficacy of intravenous H_2_-administration, and that lack of a comparison group precluded a precise determination of the efficacy of intravenous H_2_-administration. We are also aware that in this study we did not include a group comprised of patients treated only with intravenous H_2_-administration. Our study showed that the serum MDA-LDL levels on the 7th day after admission did not differ significantly from that of pretreatment in GI patients, although GII patients experienced a significant decrease in MDA-LDL levels. As for the functional outcome, the efficacy of the treatment with edaravone and H_2_-enriched intravenous solution was not obvious in this study. These findings may suggest the need for a more aggressive treatment protocol, such as more frequent daily administration of H_2_.

## Conclusions

The present study demonstrated the safety of a treatment paradigm using an H_2_-enriched intravenous solution for patients with acute cerebral infarction, including patients treated with t-PA. A prospective randomized study is warranted prior to making conclusions about the efficacy of this treatment. We are currently organizing a new study to address these limitations.

## Competing interests

The authors declare that they have no competing interests.

## Authors’ contributions

The authors equally contributed to the production of this article and have read and approved the final manuscript.
